# Alternative Approaches to Measurement of Ground Vibrations Due to the Vibratory Roller: A Pilot Study

**DOI:** 10.3390/s19245420

**Published:** 2019-12-09

**Authors:** Jan Nedoma, Martin Stolarik, Stanislav Kepak, Miroslav Pinka, Radek Martinek, Jaroslav Frnda, Michael Fridrich

**Affiliations:** 1 Department of Telecommunications, Faculty of Electrical Engineering and Computer Science, VSB—Technical University of Ostrava, 17. listopadu 15, 708 33 Ostrava-Poruba, Czech Republic; stanislav.kepak@vsb.cz (S.K.); michael.fridrich@vsb.cz (M.F.); 2 Department of Geotechnics and Underground Engineering, Faculty of Civil Engineering, VSB—Technical University of Ostrava, Ludvika Podeste 1875/17, 708 33 Ostrava-Poruba, Czech Republic; miroslav.pinka@vsb.cz; 3 Department of Cybernetics and Biomedical Engineering, Faculty of Electrical Engineering and Computer Science, VSB - Technical University of Ostrava, 17. listopadu 15, 708 33 Ostrava-Poruba, Czech Republic; radek.martinek@vsb.cz; 4 Department of Quantitative Methods and Economic Informatics, Faculty of Operation and Economics of Transport and Communications, University of Zilina, 01026 Zilina, Slovakia

**Keywords:** ground vibrations, vibratory roller, vibration measurements, interferometric sensor, fiber-optic sensor

## Abstract

At present, one of the primary tasks of the construction industry is to build transport infrastructure. This concerns both the construction of new bypasses of towns and the repair of existing roads, which are damaged by congestion, especially by freight transport. Whether it is a new building or a reconstruction, it is always very important to choose a suitable method of subsoil treatment. One of the most commonly used methods for soil treatment is currently compaction using vibratory rollers. This method is very effective both in terms of results and due to its low financial demands compared to other methods. Vibration is transmitted to the surrounding rock environment when compacting the subsoil using vibratory rollers. Although the intensity of these vibrations is not as pronounced as in other methods of subsoil treatment, such vibrations can have a significant effect, for example during compaction in urban areas or in an area with the presence of historical objects. Therefore, it is very advisable to monitor the effect of these vibrations on the environment during construction. This paper brings an original experimental comparative study of standard seismic instrumentation with a developed interferometric sensor for the field of monitoring vibrations generated during compaction of subsoil using vibrating rollers. The paper presents time and frequency domain results, as well as attenuation curves, which represent real attenuation of vibrations in a given rock environment. The results presented here show that a system operating on a different physical principle from the one used at present has the potential to replace the existing, very expensive, seismic equipment.

## 1. Introduction

Subsoil compaction with vibratory rollers has been the most commonly used building technology in recent decades. The advantages of this technology include speed and low cost [1]. However, this is a complicated process, and in order to achieve the best compaction results, the design of the compaction rollers is still in development [2,3,4,5]. One of the negative effects of this technology is the generated vibration that is transmitted to the surrounding rock environment [6,7]. These vibrations are not routinely monitored because the intensity of these vibrations is not as significant as in other dynamic subsoil methods, such as dynamic consolidation [8,9]. However, if dynamic roller compaction occurs, for example, in urban areas, in areas where historical objects are present, these vibrations can have a significant impact, as well as in building facilities where instruments that are highly susceptible to vibrations are located [10].

The vibration intensity generated by the compaction rollers reaches relatively large values over a small distance, but their attenuation is relatively fast and best corresponds to the exponential dependence of amplitude on distances [11,12,13]. However, in unfavorable geological conditions, vibrations can be transmitted over longer distances, and in these cases, standard seismic monitoring is also carried out on objects [14]. Mathematical modeling can also be used to predict the propagation of vibrations through the rock environment, but it is always advisable to verify these models by in situ seismic measurements [15,16].

With regard to the purchase price of standard seismic instrumentation, it is possible in this case to look for less expensive alternatives for monitoring dynamic effects from vibratory rollers. The presented comparative study builds on previous research into the suitability of optical fiber devices for vibration monitoring from various sources of dynamic loads. An experimentally developed interferometric sensor was used to measure rock mass dynamic response, whose applicability already has been verified for similar dynamic tasks [17,18,19]. A standard seismic station was then used to compare the results. Results of experimental measurements are presented in both the time and frequency domain. In addition, profile measurements were carried out, resulting in a graphical representation of the attenuation of the rock environment in the form of attenuation curves (dependence of amplitude on distance from the source of dynamic load). The main benefit of this study is not the introduction of a new measuring device, but the application of an experimentally tested interferometric sensor in an area where this type of sensor has not yet been applied and the presentation of the original results of the whole study.

## 2. State-of-the-Art

Article [20] describes a displacement detector for slight vibration measurement. It uses lens assemblies, a mirror, a quarter wave plate, and a polarization beam splitter. A four quadrant photodiode detects the energy change ratio of a laser beam and translates the signal into the corresponding displacement. Even slight vibration can be measured; however, the presented sensor device was constructed in the laboratory on an optical table, which is not applicable in the outdoor environment. Furthermore, the presented frequency range was limited to 7 Hz, which could be improved in the future.

Distributed fiber optic sensors are promising tools for vibration measurement, as well as for structural health monitoring [21]. It can measure physical quantities (such as vibration) continuously along the long distance fiber. The system can provide thousands of accelerometers along a single mode fiber (SMF) optic cable connected to or embedded in structures. The achievable frequency range can be up to several kHz, well above the requirements for the desired application. Several field demonstrations of distributed technology were already presented [22]. The main drawback is the price of implementation, mainly the evaluation unit.

Optical sensors based on fiber Bragg gratings (FBG) can be used to measure vibration from traffic. In [23], the authors presented accelerometers with gratings capable of measuring vibration from passing trams or trains. Sensors were placed adjacent to the rail at a 0.5–2 m distance. All passing rail vehicles were reliably captured, and the frequency response was determined, although no usable sensor frequency range was reported. Usually, the reported frequency range for grating based sensors was up to 100 Hz [24].

A polarimeter fiber vibration sensor was also reported [25]. A polarization diversified loop with a short polarization maintaining (PM) photonic crystal fiber (PCF) as a sensor head was used. The frequency response of the sensor was up to 3 kHz. Large scale deployment into practice was limited due to the use of PM fibers and passive optical splitters, which are expensive to manufacture and not compatible with existing fiber networks.

Fiber-interferometer based sensors operate on several principles with various configurations (such as Fabry–Perot, Mach–Zehnder, Sagnac, Michelson, and more) and have found applications across a wide range of civil engineering applications. Usually, scientists report a wide frequency range for this type of sensor.

In [26], a fiber-optic dual ring Michelson interferometer was proposed for the measurement of dynamic signals. It used FBGs with different central wavelengths as a refractive element. For each output beam, two interference signals with different wavelengths were obtained by a two channel wavelength division multiplexer, and a 3× coupler was used to demodulate the phase changes passively. The reported frequency range was up to 19 kHz.

A vibration sensor was also demonstrated based on strongly coupled core optical fiber [27]. The interferometer consisted of a low insertion loss multi-core fiber structure, where only two super-modes interfered. This part of the sensor was sensitive to vibration and unaffected by temperature changes. The authors demonstrated that the sensor was capable of operating in the 2–2500 Hz frequency range.

Fabry–Perot interferometers (FPI) were reported to work in the range up to 1 kHz as well [28]. A Mach–Zehnder interferometer with tapered bend insensitive fiber for vibration measurement in the range up to 500 kHz was also successfully demonstrated [29]. Interferometers have thus been proven to be a suitable measurement tool in the desired frequency range exceeding 100 Hz.

However, many publications present fiber-optic sensor principles operating only in a laboratory environment and conditions; nevertheless, some principles have found application in practice. In [30], FBGs were mounted on a transformer, and a real-time vibration monitoring system was demonstrated. FPIs were installed on a motorway bridge as vibration sensors for monitoring the traffic induced vibrations [31]. Michelson interferometers have found their place as geophones [32].

In addition to the interferometric measurement approach, it is necessary to mention approaches in the form of distributed systems like distributed strain and temperature sensing (DSTS) [33,34,35] or fiber Bragg grating sensors [36,37,38,39,40,41,42]. Both technologies work on a different principle and bring some advantages and disadvantages to the field of civil engineering, but based on our best opinion for the measurement of ground vibrations due to the vibratory roller, we chose and proposed a solution based on the fiber-optic interferometer.

The aim of the article and the proposed sensor system is to extend the application potential of fiber-optic sensors in construction engineering. Our constructed sensor worked, therefore, on the well known physical principles of the Mach–Zehnder interferometer, which have been found to be optimal for measuring vibration from compaction, where such a sensor has not been used yet. Furthermore, the sensor was made to withstand the outdoor environment and built from readily available materials, including single mode fiber passive optical components. Last but not least, the sensor is compatible with the most common type of single mode optical fibers.

## 3. Methods

### 3.1. Seismic Equipment BRS32

**Figure 1:**
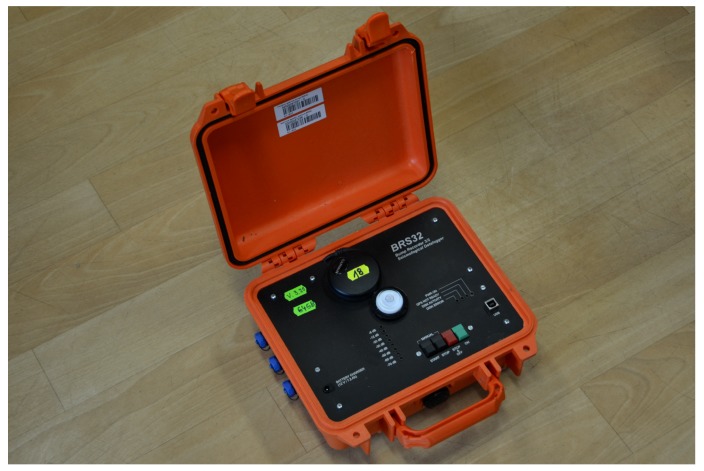
Universal seismic station BRS32.

The BRS32 is a universal seismic station applicable to all seismic measurements (Figure 1). The station is equipped with a three component seismic geophone. The frequency and dynamic range range depends on the internal geophone. It ranges from 0.5 Hz to 80 Hz at dynamics up to 120 dB. The recorder itself has an input dynamic range greater than 144 dB. Internal sensory equipment can be selected, depending on the character of the measurement, either with internal Dutch SM6 geophones (4.5 to 100 Hz), or with 1 Hz LE3D sensors from the German company Lennartz (1 to 80 Hz), or to connect another external sensor [43]. The SM6-3D was installed on the instrument used in this study [43].

### 3.2. Experimentally Developed Fiber-Optic Interferometric Sensor

The interferometer is a device that works by merging two or more sources of light to create an interference pattern, which can be measured and analyzed. This phenomenon can also be observed in optical fibers, which is a form of waveguide. Interferometers are often used because they can measure even small changes in the light, which is not achievable any other way. The interference patterns generated by interferometers contain information about the measured phenomena, in our case the vibration.

**Figure 2:**
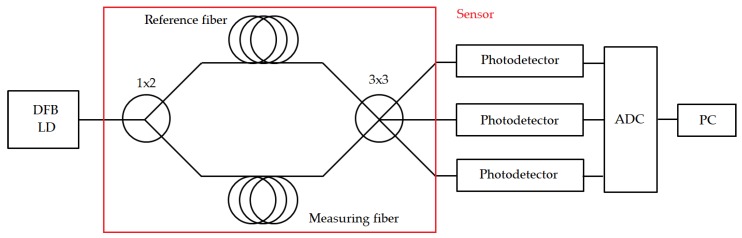
The fiber-optic interferometric measurement system diagram. DFB, distributed feedback.

Phase shifting technology has been adapted to a variety of interferometer types. For our purpose, a Mach–Zehnder interferometer was assembled. A fiber coupled light was first split into two parts by a 1 × 2 splitter. These two paths are called the reference arm and measuring arm. The optical path lengths in the two arms were nearly identical, so the interferometer was balanced. Both interferometer arms were connected to the two inputs of the second 3 × 3 coupler forming the sensor; see Figure 2. The length of the optical fiber in the interferometer arms was 3 m, including coupler pigtails.

**Figure 3:**
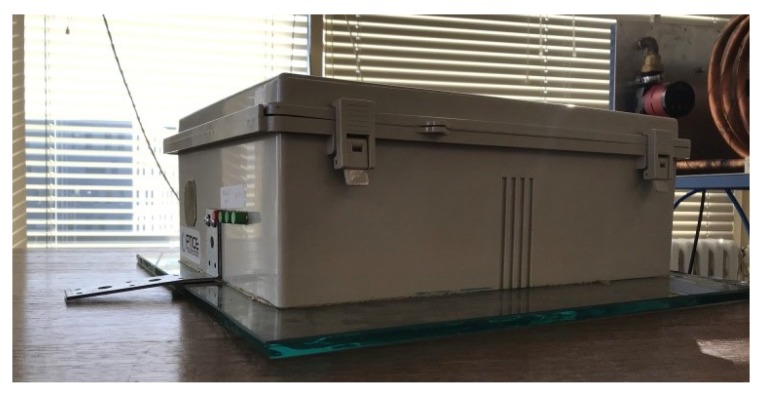
The interferometric sensor in the laboratory.

The sensor was enclosed in a plastic box with one input and three output connectors. The measuring single mode G.652.D fiber in 900 μm tight buffer was glued to the 50 × 50 × 1 cm glass base using epoxy resin, which had a similar elastic modulus to the base. The phase delay of the light passing through the measuring fiber was affected by the vibration with which the optical fiber came into direct or indirect contact. The reference fiber of the same length and optical couplers were placed between two layers of vibration insulation material (glass wool was used in this experiment); the sensor is depicted in Figure 3. In accordance with ISO 4866: 2010, the sensor was sensitive in the frequency range from 1 to 300 Hz.

As the radiation source, a 1540 nm distributed feedback (DFB) laser diode (LD) was used. These types of lasers with high coherence lengths provided light to make the interferometer balanced. The optical photodetectors were custom-made and consisted of regular InGaAs p-i-n pigtailed photodiodes and amplifying electrical circuits. The optical fiber connecting the laser and the photodetectors could be very long (up to several kilometers) because of the phase based measurement principle.

For the analog voltage-to-digital signal conversion, the National Instruments 9239 in cDAQ-9171 chassis data acquisition module was used. Having four 24-bit analog inputs with a sampling frequency up to 50 kS/s, the module provided precision and stable signal conversion to the computer using the universal serial bus (USB). The computer and LabVIEW application were used to collect sensor signals and process the data. Data processing included namely the passive phase demodulation, which was based on the three signals produced by the 3 × 3 coupler and the use of the digital arctangent function described in [30].

## 4. Experimental Setup

Experimental comparative measurement was carried out during the construction of the bypass of the city of Krnov in the Czech Republic. Two bridges will also be built in the bypass, and measurements were made during the building of the transitional region of the western bridge (GPS coordinates 50.1046819 N, 17.6632819 E). The soil body of the transitional region was built in layers 0.5 m thick. The measurement was carried out during the construction of the twelfth layer. The material for the embankment was crushed gravel with a 0/32 fraction. In the subsoil of the embankment, there was fine grained soil F6 of a clay-like character located at a depth of up to 2.9 m; from 2.9 to 3.9, there was gravel with an admixture of fine grained particles G3; and below that, up to a depth of 5 m, there was weathered rock characterized as class G4/R6. Up to a depth of 15 m, where the drilling operations were finished, the subsoil then formed a solid rock of class R3/R2. The groundwater level was not reached during drilling.

**Figure 4:**
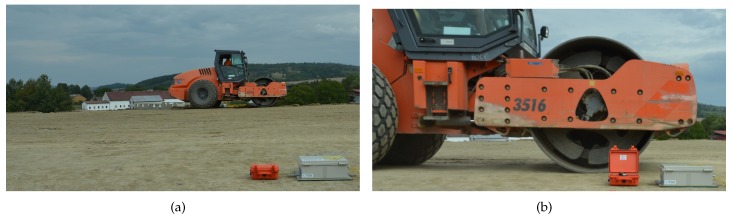
Photo documentation taken during the comparative measurement: (**a**) maximum distance of the vibrating roller from the measuring equipment: 13.5 m; (**b**) the minimum distance of the vibrating roller to the measuring equipment: 1.4 m.

**Figure 5:**
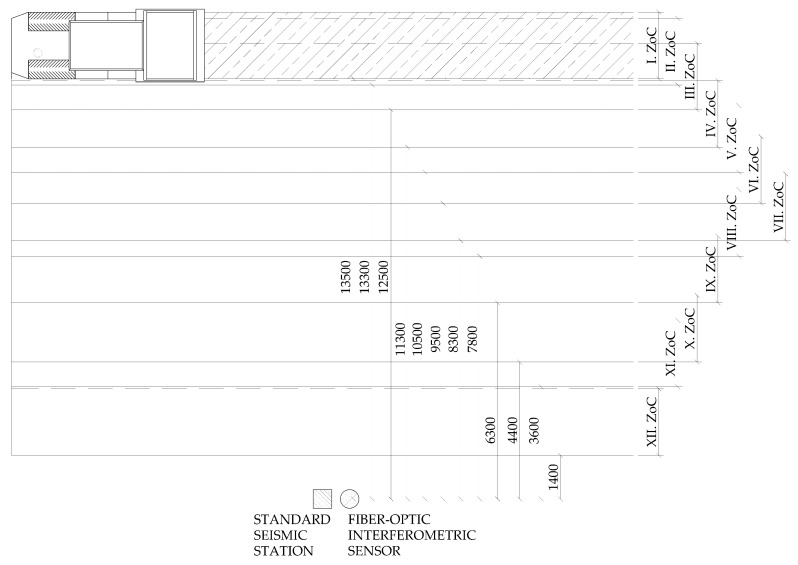
Scheme of the whole measurement during compaction. ZoC, zone of compaction.

Both the seismic station and the interferometric sensor were situated in one place during the whole measurement, and the vibrating roller compacted individual strips of soil (a total of twelve zones of compaction (ZoC)) at different distances from the sensors (Figure 4 and Figure 5). This distance ranged from 1.4 m to 13.5 m; please see Figure 5. The compaction procedure was carried out with a Hammtronic HAMM H 11i. The HAMM H 11i is a single drum roller with a vibration trowel weighing 10,880 kg. The tread diameter was 1504 mm, and the length was 2140 mm. The vibration frequency could be set in the range of 20–36 Hz and the vibration amplitude of the roller in the range of 2.04–0.84 mm. During compaction, one way vibration-free travel was performed first, and then, it traveled back with vibration, first with maximum vibration (high vibrations (HV)); after compacting the entire area, it continued with medium vibrations (MV); and at the end, runs with low vibrations (LV) were made in individual zones. The amplitude setting on the roller corresponded to the following values: HV = 1.8 mm, MV = 1.5 mm, and LV = 1.3 mm. The frequency setting on the roller was then as follows: HV = 20 Hz, MV = 25 Hz, and LV = 20 Hz.

### Results

This section summarizes the results of the entire experimental comparison measurement. For better visual comparison, the graphical representation of the time and frequency domain is organized as the interferometric sensor always being below the seismic station for each of the three compaction stages with different vibration intensities. The standard seismic station, in terms of the device function, registered vibrations in three perpendicular directions. In this pilot study, only the vertical direction was selected for comparison, taking into account the nature of the vibrations generated. The time recording of the vertical direction with respect to its course also best correlated with the time recording of the interferometric sensor.

**Figure 6:**
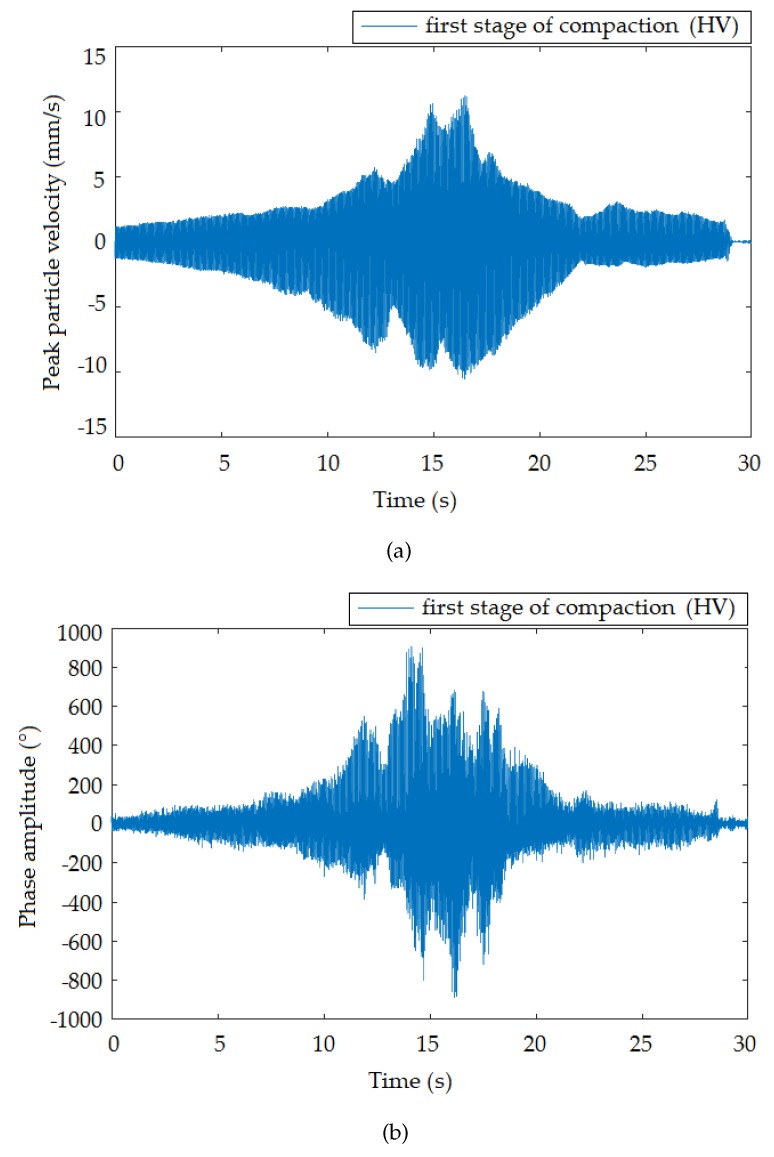
Time recording of the passage of the roller at a distance of 1.4 m from the measuring equipment: high vibrations (HV). (**a**) Seismic station; (**b**) interferometric sensor.

**Figure 7:**
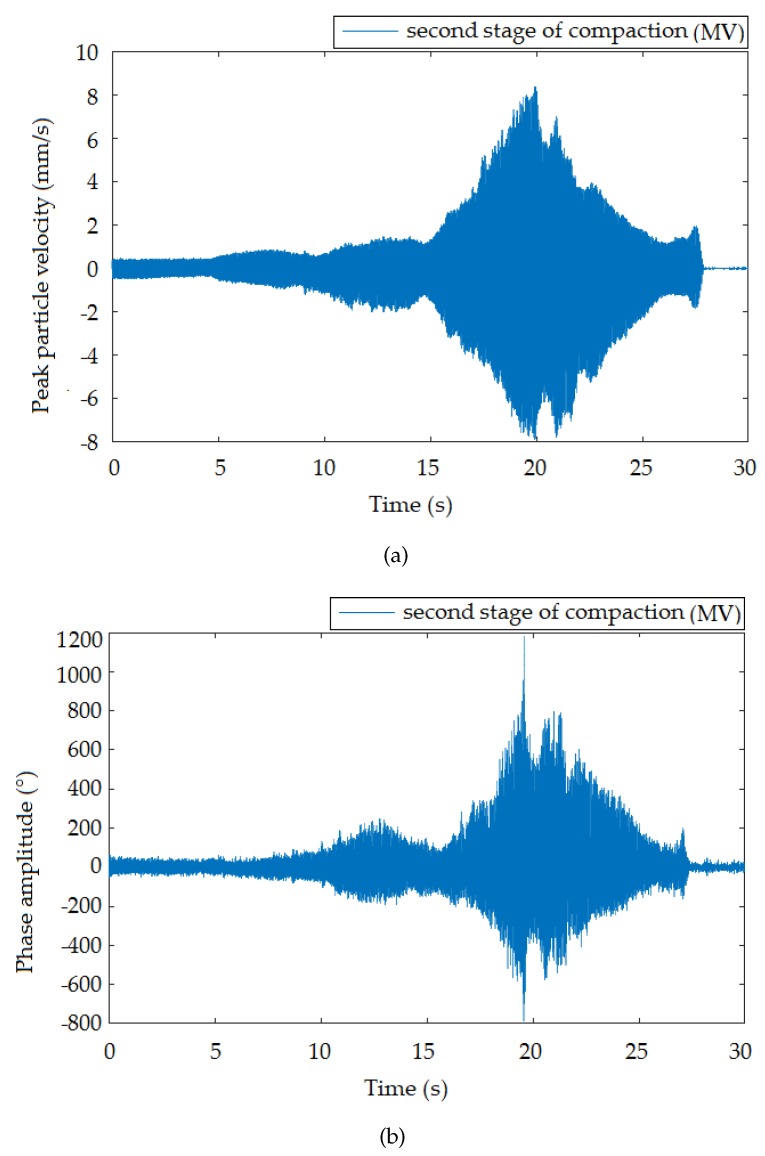
Time recording of roller passage at a distance of 1.4 m from the measuring equipment: medium vibrations (MV). (**a**) Seismic station; (**b**) interferometric sensor.

**Figure 8:**
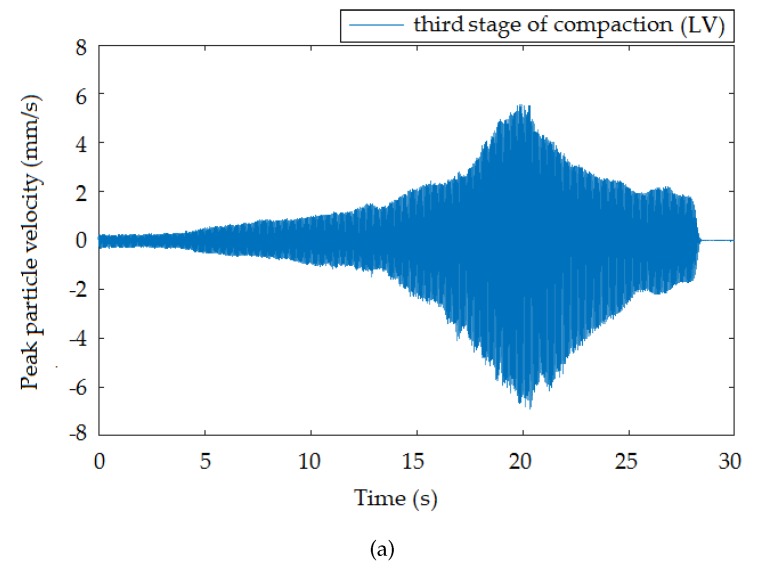

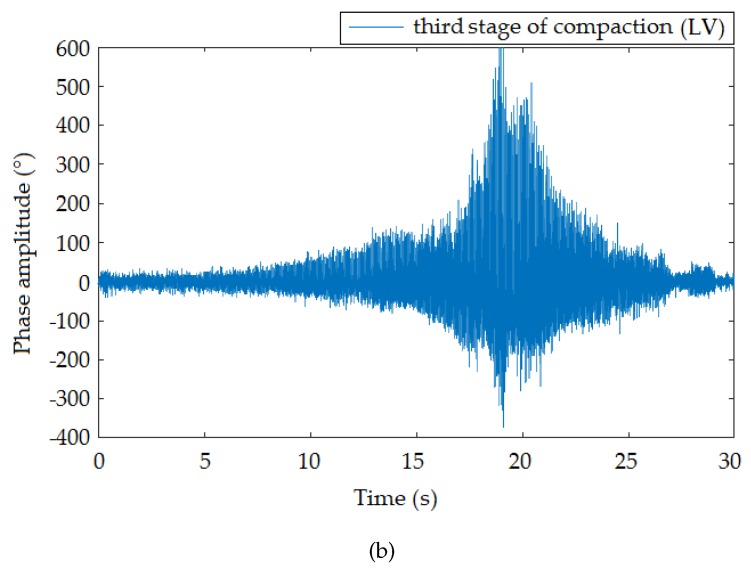
Time recording of the passage of the roller at a distance of 1.4 m from the measuring devices: low vibrations (LV). (**a**) Seismic station; (**b**) interferometric sensor.

Figure 6, Figure 7 and Figure 8 presents the time records of individual compaction intensities (HV, MV, LV) that were obtained when the vibration roller was moved at the smallest distance from the measuring devices (the smallest distance was important because it offered the most representative data representing a given dynamic load). Therefore, the data were not significantly affected and distorted by the rock environments. These data can be used for subsequent numerical analysis and processing of mathematical models for the prediction of vibration propagation. Image (a) corresponds to the time record made by the seismic station and Image (b) by the interferometric sensor. The manifestation of vibration compaction on time recordings had the typical character of this type of dynamic load [44] and was equally long for both devices. Furthermore, the recorded maxima amplitudes were noticeable always at the same time on the x-axis. These results present a good and important background for future research.

**Figure 9:**
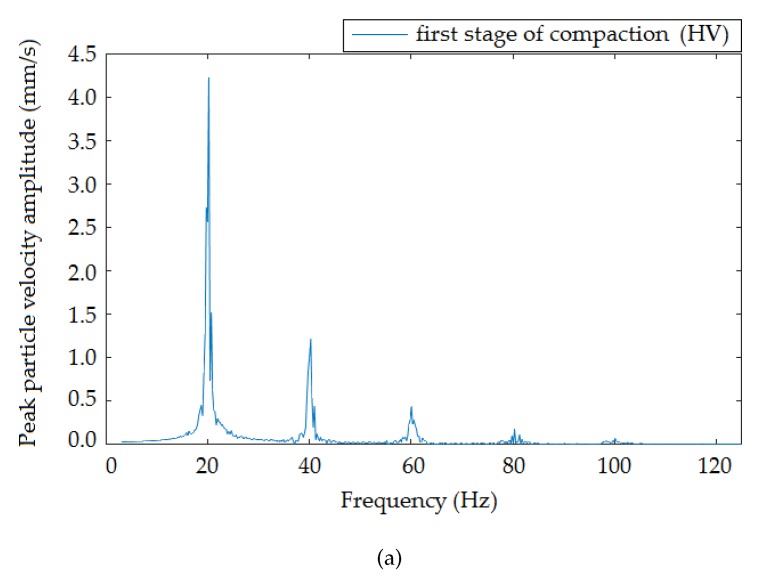

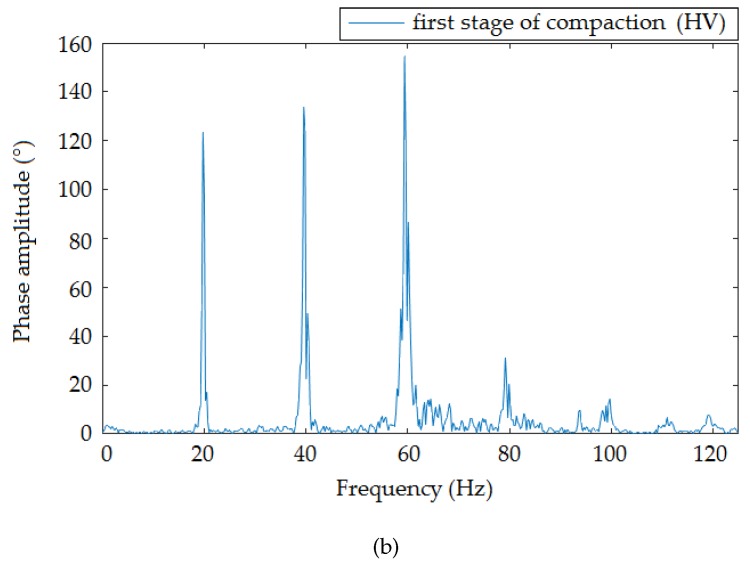
Frequency image of the passage of the roller at a distance of 1.4 m from the measuring equipment: high vibrations. (**a**) Seismic station; (**b**) interferometric sensor.

**Figure 10:**
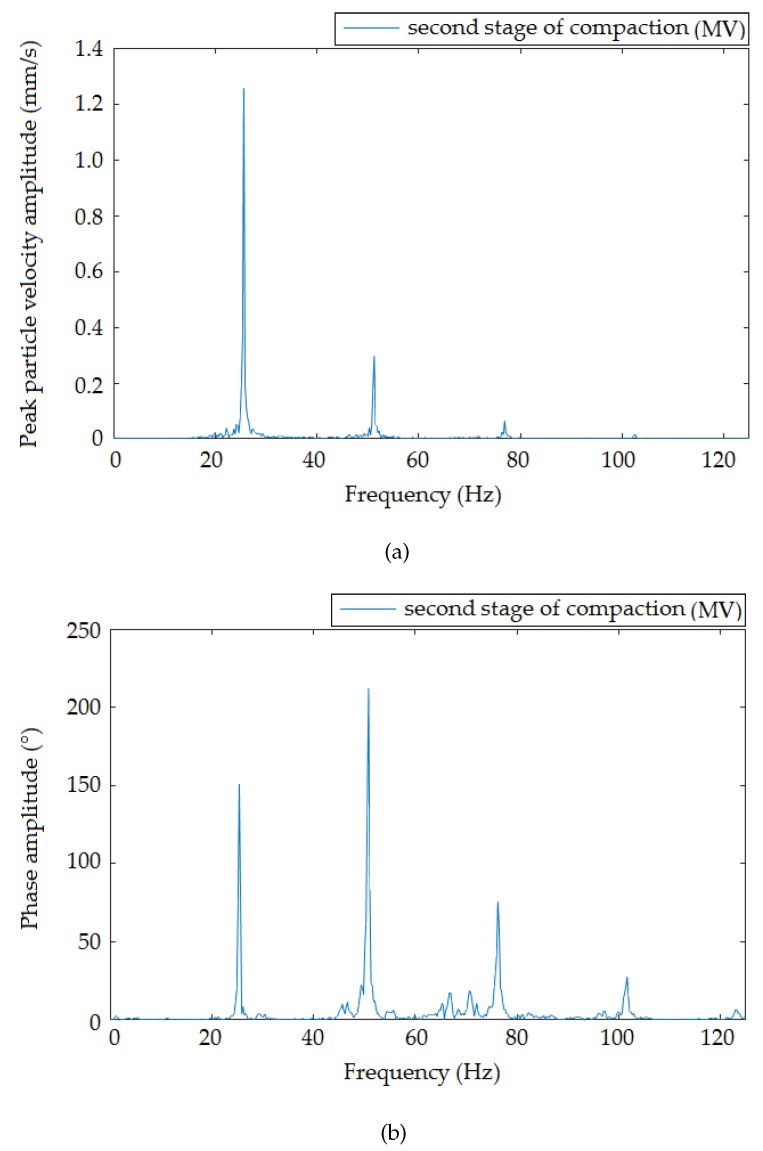
Frequency image of the passage of the roller at a distance of 1.4 m from the measuring equipment: medium vibrations. (**a**) Seismic station; (**b**) interferometric sensor.

**Figure 11:**
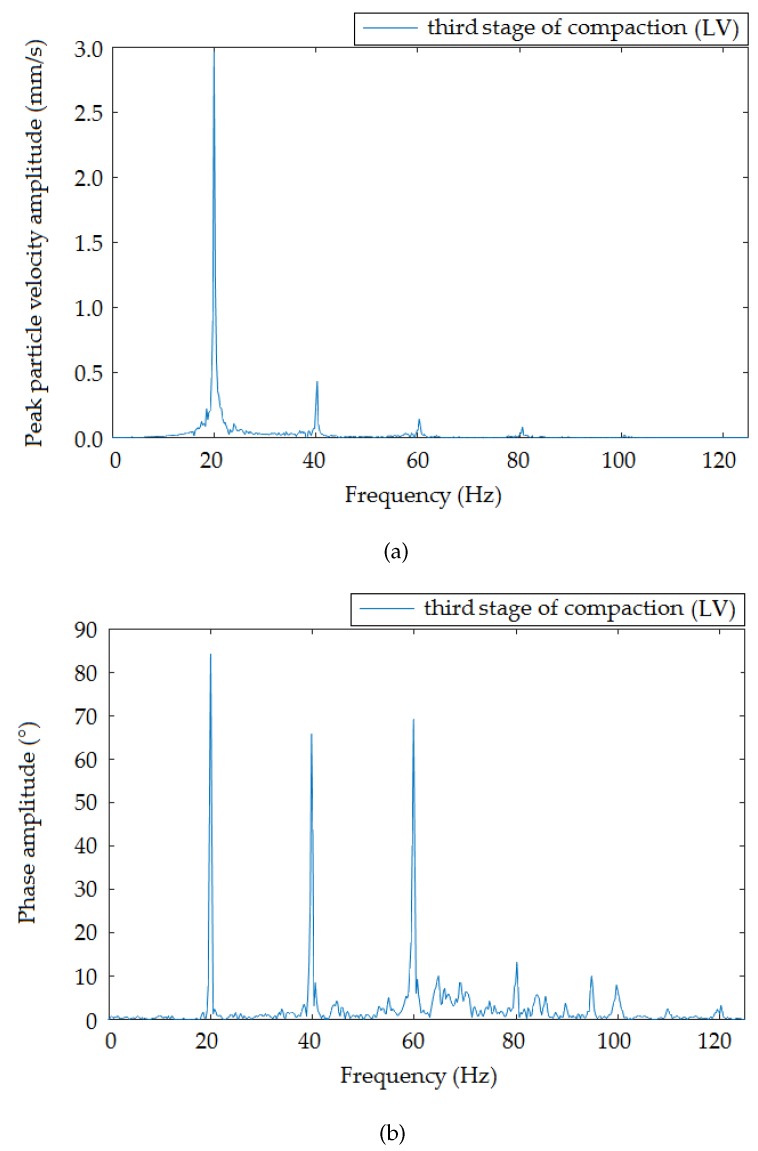
Frequency image of the passage of the roller at a distance of 1.4 m from the measuring equipment: Low vibrations. (**a**) Seismic station; (**b**) interferometric sensor.

Figure 9, Figure 10 and Figure 11 present the frequency spectra of individual compaction stages (HV, MV, LV) that were recorded when the roller moved at the smallest distance from the measuring devices. The results recorded by the interferometric sensor corresponded to the frequency spectrum recorded by the seismic station. In all spectra, there was a distinct peak in Frequencies 20 (Figure 9), 25 (Figure 10), and 20 (Figure 11) Hz, which were the frequencies corresponding to the compaction frequency of the setting on the vibratory roller, where the following peaks corresponded to multiples of this frequency.

**Figure 12:**
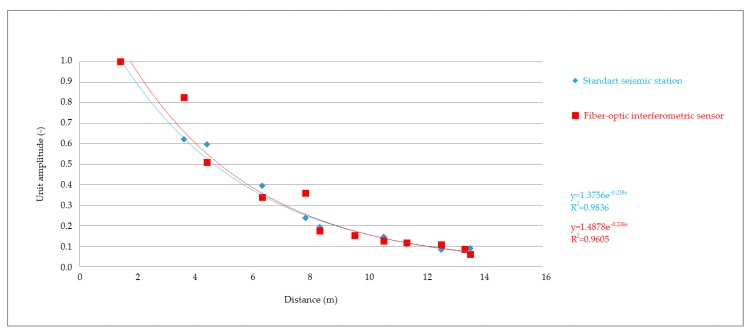
Attenuation curves. Comparison of the seismic station and interferometric sensor: high vibration.

**Figure 13:**
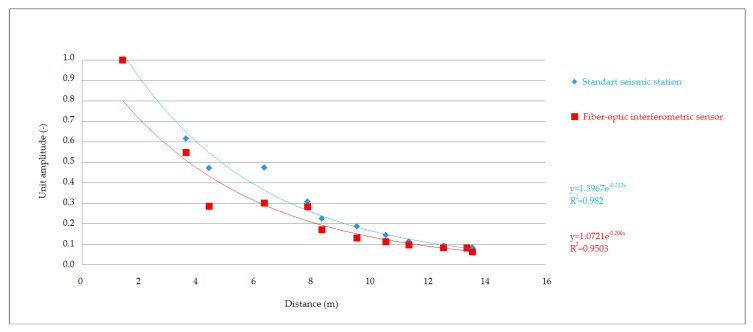
Attenuation curves. Comparison of the seismic station and interferometric sensor: medium vibrations.

**Figure 14:**
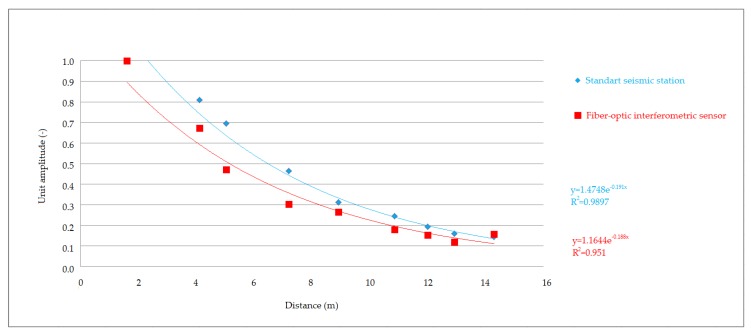
Attenuation curves. Comparison of the seismic station and interferometric sensor: low vibrations.

The recordings made at all stages and compaction intensities were very similar to each other, both in the time and frequency domain. This led to the conclusion that the whole set of data from the performed comparative measurement could be used for graphical representation of vibration attenuation in the rock environment in the form of attenuation curves. Significant changes in the recorded data would probably indicate significant changes in local geology or registration of various types of seismic waves. Figure 12, Figure 13 and Figure 14 show the attenuation curves, which were obtained from the data collected by the seismic station and the interferometric sensor. The unit amplitude values are plotted on the vertical axis of the graph, and the attenuation is plotted as the exponential dependence of this amplitude on the distance from the source of vibration. The resulting attenuation curves were very similar: they had a high correlation coefficient, and the exponents in the attenuation equation showed a significant agreement.

## 5. Discussion

The vibrations generated during compaction of soils by vibrating rollers generally are classified as vibrations of so-called technical seismicity, i.e., anthropogenic vibrations. The basic aim in monitoring these anthropogenic vibrations is to monitor the impact on the surrounding development in close proximity to the construction activity generating vibrations and to determine the level of attenuation in the rock environment. The evaluation of measured data is usually performed in the amplitude and frequency range, and the obtained maximum amplitude and frequency values are evaluated based on, e.g., national standards [45,46,47,48]. All seismic monitoring is currently carried out using piezoelectric seismometers, which are standard, but relatively expensive, measuring devices. Our pilot study showed that it was possible to find an alternative and progressively developing method utilizing an optical interferometer for measuring seismic effects, e.g., due to vibrating rollers.

The first results presented showed an extraordinary consistency in both the time and frequency domain. In the time domain, all the maximums and lengths of the individual records matched. The attenuation curves for the given rock environment were also computed from the maxima, when converted to unit amplitude, and both the dependence of the amplitude on distance obtained from the values measured by the seismic station and the same dependence obtained from optical interferometer measurements showed a high correlation coefficient R2 $\dot{-}$ 0.95 – 0.98. Slightly greater variance was evident in the graphs for the experimentally developed interferometer, which was due to the pilot measurement, and greater consistency can be achieved by proper calibration. Similarly, the exponents in the attenuation equation showed a concordance of the two devices, the differences being in the thousandths.

It was possible to identify identical peaks in the frequency spectra that corresponded to the compaction frequency of the vibrating roller and its multiples. In spectra taken from the seismic station, these multiples were exponentially attenuated (correlation coefficient R2 $\dot{-}$ 0.98 for all three compaction intensities). This was not the case in the spectra from an interferometric sensor, which was given by both the measurement method (MZ interferometer) used and the sensor design itself, where the sensitivity of this experimentally developed sensor was not yet linear.

A major drawback of the interferometric sensor was its current design, which so far does not allow us to measure in three perpendicular directions, as the standard seismic sensor allows, but at present, such a full fledged three component interferometric sensor in our country is being developed and laboratory tested. Similarly, this interferometric sensor has not yet been calibrated, and the conversion to commonly used physical quantities (speed/rate or acceleration) in the field of seismic and geotechnical engineering has not been processed.

Although the interferometer is one of the most sensitive fiber-optic sensor principles, this does not automatically constitute sensor operation readily. Multiple interferometer configurations are known; however, not all of them are suitable for presented measurement. Considering the cost effectiveness of the proposed device, a Mach–Zehnder interferometer is a good option (Michelson interferometers require additional mirrors, etc.). Even then, the operational sensor frequency range and sensitivity are determined mainly by the proper sensor construction of both measurement and reference optical paths. In this paper, we presented the possibility to make such a custom-designed sensor operational at decent costs acceptable by end-users. Furthermore, in this paper, we present the application of an experimentally tested interferometric sensor in an area where this type of sensor has not yet been applied and the presentation of the original results of the whole study.

Below (Table 1), we present a comparison of the basic parameters of a conventional seismic station and the proposed interferometric sensor (including interrogator units). The price in the case of an interferometric sensor is only approximate (the price was calculated based on the choice of individual components that were purchased individually).

## 6. Conclusions

The paper presented a unique comparative pilot study of the application of an experimentally developed interferometric sensor and standard instrumentation in the field of geotechnical engineering. In this study, we monitored the dynamic response of the rock environment and vibration attenuation from the vibratory roller used to compact the subsoil of the road.

The measurements were carried out at distances from 1.4 m to 13.5 m from the vibratory roller within three different compaction modes. In the time domain, there was an unequivocal match in all records when comparing the interferometric sensor and the seismic station both in the length of individual records and in the identification of maxima. In the frequency domain, individual peaks were clearly detected corresponding to the roller compaction frequency. The resulting attenuation curves were very similar, and they had a high correlation coefficient, while the exponents in the attenuation equation showed a significant agreement.

Our pilot study showed that it was possible to find a cost effective and alternative method utilizing fiber-optic technology for measuring seismic effects caused by vibrating rollers.

## Figures and Tables

**Table 1:** Comparison of basic parameters of the proposed interferometric sensor and used universal seismic station BRS32.

Type of Sensor	Frequency Range (Hz)	Size (mm)	Weight (kg)	Price (USD)	Sampling Frequency (Hz)
BRS32	4.5–100	270 × 245 × 130	4.6	3100	125–500
Interferometric	1–5000	500 × 500 × 350	7.8	1600	250–50,000
